# Deep RNA Sequencing Reveals Hidden Features and Dynamics of Early Gene Transcription in *Paramecium bursaria* Chlorella Virus 1

**DOI:** 10.1371/journal.pone.0090989

**Published:** 2014-03-07

**Authors:** Guillaume Blanc, Michael Mozar, Irina V. Agarkova, James R. Gurnon, Giane Yanai-Balser, Janet M. Rowe, Yuannan Xia, Jean-Jack Riethoven, David D. Dunigan, James L. Van Etten

**Affiliations:** 1 Laboratoire Information Structurale and Génomique UMR7256 CNRS, Aix-Marseille Université, Marseille, France; 2 Department of Plant Pathology, University of Nebraska, Lincoln, Nebraska, United States of America; 3 Nebraska Center for Virology, University of Nebraska, Lincoln, Nebraska, United States of America; 4 Center for Biotechnology, University of Nebraska, Lincoln, Nebraska, United States of America; The Ohio State University, United States of America

## Abstract

*Paramecium bursaria* chlorella virus 1 (PBCV-1) is the prototype of the genus *Chlorovirus* (family *Phycodnaviridae*) that infects the unicellular, eukaryotic green alga *Chlorella variabilis* NC64A. The 331-kb PBCV-1 genome contains 416 major open reading frames. A mRNA-seq approach was used to analyze PBCV-1 transcriptomes at 6 progressive times during the first hour of infection. The alignment of 17 million reads to the PBCV-1 genome allowed the construction of single-base transcriptome maps. Significant transcription was detected for a subset of 50 viral genes as soon as 7 min after infection. By 20 min post infection (p.i.), transcripts were detected for most PBCV-1 genes and transcript levels continued to increase globally up to 60 min p.i., at which time 41% or the poly (A+)-containing RNAs in the infected cells mapped to the PBCV-1 genome. For some viral genes, the number of transcripts in the latter time points (20 to 60 min p.i.) was much higher than that of the most highly expressed host genes. RNA-seq data revealed putative polyadenylation signal sequences in PBCV-1 genes that were identical to the polyadenylation signal AAUAAA of green algae. Several transcripts have an RNA fragment excised. However, the frequency of excision and the resulting putative shortened protein products suggest that most of these excision events have no functional role but are probably the result of the activity of misled splicesomes.

## Introduction

Viruses in the family *Phycodnaviridae*, together with those in the *Poxviridae*, *Iridoviridae*, *Ascoviridae*, *Asfarviridae*, and the *Mimiviridae* are believed to have a common evolutionary ancestor and are referred to as nucleocytoplasmic large DNA viruses (NCLDV) [1]–[3]. Members of the *Phycodnaviridae* consist of a genetically diverse, but morphologically similar, group of large dsDNA-containing viruses (160 to 560 kb) that infect eukaryotic algae [4], [5]. These large viruses are common in both terrestrial and marine waters throughout the world.

*Paramecium bursaria* chlorella virus 1 (PBCV-1), the prototype of the *Phycodnaviridae* family (genus *Chlorovirus*), is a large, icosahedral (190 nm in diameter) virus that infects the unicellular, eukaryotic green alga *Chlorella variabilis* NC64A. PBCV-1 has a 331-kb linear double-stranded DNA (dsDNA) genome with covalently closed hairpin ends and contains 416 predicted protein-encoding sequences (CDSs) as well as 11 tRNA encoding genes [6]. A proteomic study revealed that the purified virion is associated with 148 unique virus-encoded proteins (about 35% of the virus coding capacity) and 1 host protein [6]. Approximately 40% of the 416 PBCV-1 gene products resemble proteins in the public databases.

PBCV-1 initiates infection by attaching rapidly to the cell wall of its host [7], at a unique virus vertex [8], [9]. Attachment to its host receptor is a major factor in limiting the host range. This event is immediately followed by host cell wall degradation by one or more virus-packaged enzymes at the point of contact. After wall degradation, the viral internal membrane presumably fuses with the host membrane, causing host membrane depolarization [10], potassium ion efflux [11], and an increase in the cytoplasmic pH [12]. These events are predicted to facilitate entry of the viral DNA and virion-associated proteins into the cell [13]. Host membrane depolarization also inhibits many host secondary transporters [12] and probably prevents infection by a second virus [14]. PBCV-1 lacks a gene encoding a recognizable RNA polymerase or a RNA polymerase subunit, and RNA polymerase activity was not detected in PBCV-1 virions [6]. Therefore, viral DNA and virion-associated proteins are predicted to migrate to the nucleus. Early viral transcription is detected 5 to 10 min post infection (p.i.), presumably by commandeering a host RNA polymerase(s) (probably RNA polymerase II) [15]. This rapid initiation of virus transcription suggests that some component(s) facilitates active transport of virus DNA to the nucleus. Virus DNA synthesis begins 60 to 90 min p.i., followed by virus assembly at 3 to 5 h p.i. in localized regions of the cytoplasm, called virus assembly centers [16]. At 6 to 8 h p.i., virus-induced host cell lysis occurs resulting in release of progeny virions (∼1,000 virus particles/cell, ∼25% of which form plaques) [17].

A microarray study of poly(A+)-containing RNA using *C. variabilis* cells infected by PBCV-1 showed that the PBCV-1 replication cycle is temporally programmed and regulated [18]. 227 genes were expressed before virus DNA synthesis began. These 227 CDS were grouped into two classes: 127 transcripts disappeared prior to initiation of virus DNA synthesis (considered early), and 100 transcripts were still detected after virus DNA synthesis began (considered early/late). One hundred and thirty three of the CDSs were expressed solely after initiation of virus DNA synthesis (considered late). To initiate PBCV-1 transcription, the host RNA polymerase(s), possibly in combination with a virus transcription factor(s), must recognize virus DNA promoter sequences. Three short nucleotide sequences were identified in suspected virus promoter regions (150 bp upstream and 50 bp downstream of the ATG translation start sites) that were conserved in PBCV-1 and other *Chlorovirus* members [19]. In general, PBCV-1 ORFs are not spatially clustered on the genome by either temporal or functional class, suggesting that transcriptional regulation may occur via cis- and trans-acting regulatory elements.

To understand the global dynamics of PBCV-1 gene expression during the early phase of infection, a RNA-seq study of *C. variabilis* cells infected by PBCV-1 was conducted. cDNAs from poly(A+)-containing RNAs isolated from cells at six time points covering the first hour of infection (i.e., before virus DNA synthesis begins) were sequenced using an Illumina sequencer. A total of 105 million 50-bp reads were generated and mapped to both the host and virus genomes [6], [20]; these data were used to measure the abundance of mRNAs. Here we address three significant issues regarding viral transcription. i) What are the sequential events of viral gene activation? ii) Do viral genes use host-like poly adenylation signals? iii) Are viral mRNAs spliced? Analysis of the *C. variabilis* transcripts using the same datasets is presented in a companion paper [21].

## Results and Discussion

### Genome-wide PBCV-1 Transcriptome Map

**Figure 1:**
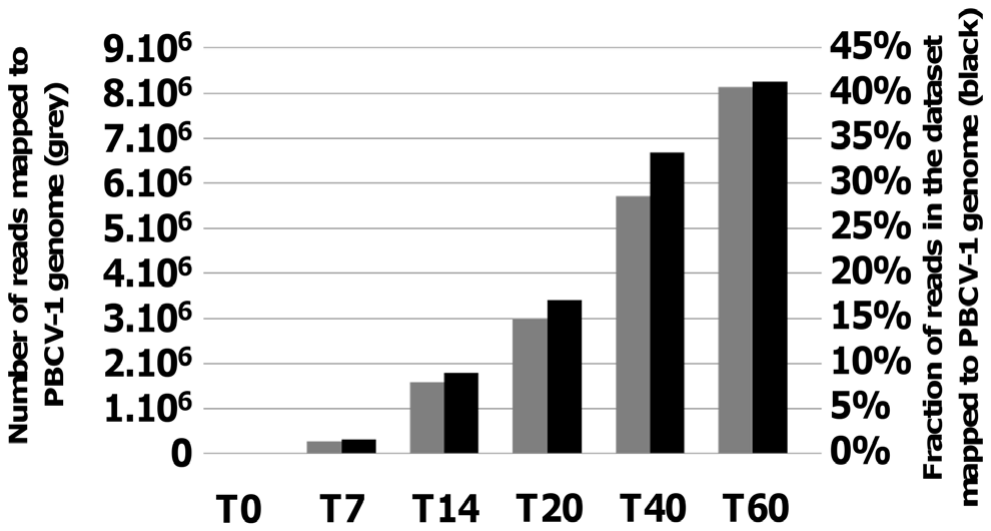
Distribution of reads mapped on the PBCV-1 genome. The grey bar is the number of reads mapped to the PBCV-1 genome and the black bar is the percentage of the total reads that map to the PBCV-1 genome.

The number and proportion of poly(A+)-containing mRNA reads were mapped to the PBCV-1 genome for each dataset corresponding to time points at 0, 7, 14, 20, 40, and 60 min p.i. (hereafter referred to as T0, T7, T14, T20, T40 and T60, respectively) (Fig. 1). No viral transcripts were detected in the T0 dataset indicating that viral mRNAs are not packaged in PBCV-1 virions. However, virus mRNAs were detected as early as T7, which indicates a rapid reprogramming of the host transcription and processing machinery to produce viral mRNAs. During the first hour of PBCV-1 infection, there was a rapid increase in the proportion of viral mRNAs. At T60, 41% of the reads mapped to the virus genome, which is in the same range as some other NCLDVs – e.g., 25–55% for vaccinia virus, see [22].

**Figure 2:**
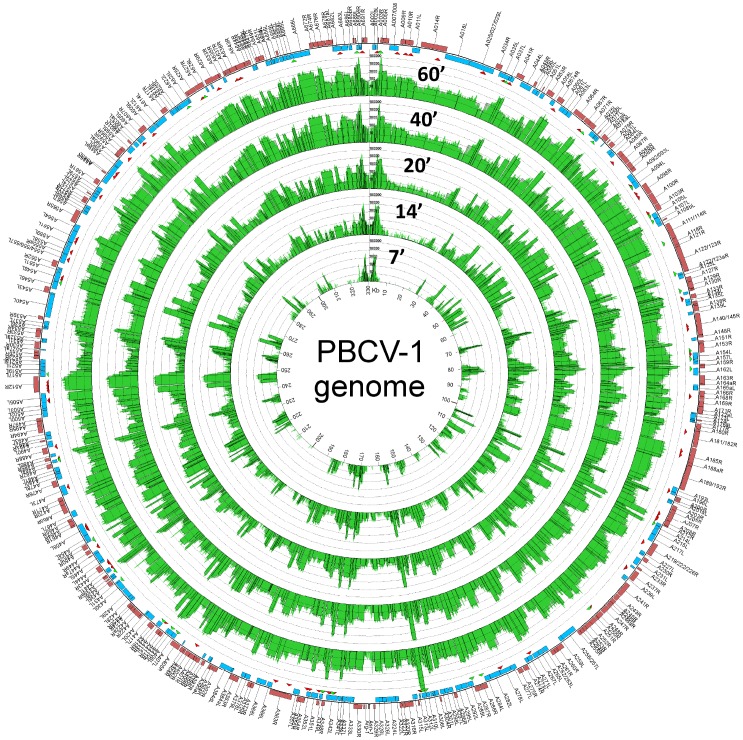
Mapping of the PBCV-1 transcriptome. PBCV-1 genes on the forward and reverse strands are depicted by red and blue boxes respectively. The green curves in the interior concentric circles represent the normalized read coverage for each time point (7, 14, 20, 40, and 60 min p.i.) of the experiment in logarithmic scale (base 10). Open boxes are superimposed onto the read coverage curves to show calculated MRPN values for each gene. Below the outer circle, red and green triangles indicate the location of predicted splice events and polyadenylation cleavage site, respectively. Note: the PBCV-1 genome is a linear molecule with inverted repeats and closed hairpin ends that is depicted as a circle. The two ends are at the 12 o’clock position.

The positions of aligned RNA reads on the virus genome were used to build single-base-resolution maps of the PBCV-1 transcriptomes. The normalized read counts along with the annotated ORFs for each of the 5 time points after infection are reported in Fig. 2. At T7 (innermost circle), the detected transcripts were restricted to a subset of PBCV-1 genes; i.e., 93% of the viral reads originated from 50 genes. These genes are probably under the control of transcription factors that are immediately active upon entry into the cell, being either a part of the host proteome or released from the virion. Remarkably the ten most highly expressed PBCV-1 genes at T7 had normalized Median Reads per-Nucleotide (MRPN) values ranging from 590 to 1,241 (MRPN is an aggregated measure of transcript accumulation similar in principle to RPKM, see Materials and Methods, Table S1). These transcript levels are comparable to host genes known to be highly expressed in cellular organisms; e.g., cytoplasmic ribosomal protein genes typically have normalized MRPN values between 600 and 2500 in the same dataset (Table S1).

Subsequently, the number of detectable PBCV-1 mRNAs increased dramatically (Fig. 1 and 2). At 60 min p.i., transcripts from virtually all of the PBCV-1 genes were detected, albeit with different intensities (Fig. S1A). For example, the mRNA level for the *a312L* gene (similar to the *Ostreococcus* virus unknown protein OsV5_171r) reached a normalized MRPN value of 165,053 (Table S1). This level of transcript accumulation is 37 times more than the most highly expressed host gene, *rbcS*, which encodes the ribulose-bisphosphate carboxylase small chain (MRPN = 4,400 at T60). Twelve other PBCV-1 genes, encoding thymidylate synthase and 11 unknown proteins, also had transcript levels higher than the *rbcS* gene in the T60 dataset. Thus the transcriptome map revealed important features of viral transcription activity during the first hour of infection: i) the sequential initiation of the virus gene transcription implies an orchestrated regulation of gene expression. ii) The RNA-seq data indicates that the levels of transcript accumulation for some virus genes are globally much higher than host genes (Fig. S1B). iii) 99% of the virus genome sequence was covered by RNA reads at T60 (Fig. 2), indicating that untranscribed viral regions are very short. This finding agrees with earlier genomic studies revealing that PBCV-1 contains many overlapping open reading frames (ORFs) [23]. Furthermore, experimental studies indicate that transcriptional read-through RNAs occur over consecutive (even non-overlapping) ORFs in PBCV-1 [24], [25] and chlorovirus CVK2, a close relative of PBCV-1 [15].

### Immediate Early Genes

The 50 most actively transcribed genes at T7 encode various functions (Table 1). They include orthologs of 18 of the 22 immediate early genes identified in chlorovirus CVK2, which also infects *C. variabilis* [15]. PBCV-1 orthologs for three CVK2 immediate early genes (*a241R*, *a342L*, and *a456L*) are not included in the top-50 T7 transcribed gene list, but were classified as early genes in a microarray study [18]. However, the earliest time point in the microarray study was 20 min p.i., a time when all 3 of these genes were expressed in the current study. The remaining CVK2 immediate early gene, that encodes a chitin synthase, has no ortholog in the PBCV-1 genome. The most actively transcribed PBCV-1 genes at T7 included at least five CDSs potentially involved in viral gene expression (RNAse III, a SWI/SNF family helicase, transcription factor TFIIB and mRNA capping enzyme alpha and beta chains). A pyrimidine dimer DNA glycosylase gene was also transcribed immediately, probably to help in DNA repair before replication of the virus genome begins [26], as well as a putative tRNA(Ile)-lysidine synthase involved in loading tRNAs with the amino acid isoleucine. Also of note, a serine/threonine-protein kinase gene was expressed early and might activate host regulation pathways at the onset of virus infection.

**Table 1:** Members of the top-50 PBCV-1 genes transcribed during the first 7 min p.i. that have a known function.

Protein id	COG functional category	Putative function
A094L	Cell Wall Degradation	Beta-1,3-glucanase
A050L	DNA Replication, Recombination, and Repair	Pyrimidine dimer-specific glycosylase
A554/556/557L	tRNA processing	tRNA(Ile)-lysidine synthase
A539R	Integration and Transposition	GIY-YIG endonuclease
A200R	Nucleotide Metabolism	Cytosine deaminase
A604L	Protein Synthesis, Modification, and Degradation	Zn metallopeptidase
A248R	Signaling	Ser/Thr protein kinase
A098R	Sugar Metabolism & Manipulation	Hyaluronan synthase
A219/222/226R	Sugar Metabolism & Manipulation	Cellulose synthase
A548L	Transcription	SWI/SNF helicase
A103R	Transcription	mRNA guanylyltransferase alpha chain
A449R	Transcription	mRNA capping enzyme beta chain
A107L	Transcription	Trancription factor TFIIB
A464R	Transcription	RNAse III

Unexpectedly the immediate-early gene set encodes 3 proteins involved in glycan metabolism pathways (beta 1,3 glucanase, cellulose-like synthase and hyaluronan synthase). Previous studies showed that transcription of the PBCV-1 hyaluronan synthase gene begins within 10 min p.i. and ends at 60–90 min p.i.; the enzyme is responsible for the accumulation of hyaluronan hair-like fibers on the external surface of the infected *C. variabilis* cell wall [27]. However, the biological function of this hyaluronan coat is unknown. The cellulose synthase-like protein may also be involved in building these hair-like fibers. Surprisingly, the cellulose synthase-like protein, a SNF2 family DNA/RNA helicase and three other immediate early gene proteins of unknown function are packaged in the PBCV-1 virion [6] (Table S2). It is possible that the virus ensures immediate availability of these proteins by both very early gene expression and packaging their protein products in the virion.

Promoter regions containing an AATGACA motif were reported to be associated with early gene expression in PBCV-1 [19]. A similar motif, ATGACAA, occurs in the promoter regions of the chlorovirus CKV2 immediate early genes [15]. In addition, two other significantly overrepresented motifs (e.g., ARNTTAANA and GTNGATAYR) were identified in PBCV-1 promoter regions but their prevalence was not associated with any temporal class of genes [19]. We examined the promoter regions (150 bp upstream and 50 bp downstream of the ATG translation start site) of the 50 most actively transcribed genes at T7 and found that AATGACA was the most prevalent motif. However, this motif was only detected in 23 (46%) of the 50 genes. These results indicate that if the AATGACA motif is a functional regulatory signal sequence, it is not the only regulatory signal sequence responsible for PBCV-1 immediate early gene expression. The two other motifs ARNTTAANA and GTNGATAYR were respectively identified in only 4 and 3 of the 50 most actively transcribed gene promoters.

Close examination of Fig. 2 suggests that immediate-early genes expressed at T7 might be spatially clustered in the genome. This hypothesis was tested by computing the average minimal distance between successive top-50 T7 transcribed genes and comparing this value with one obtained after randomizing the gene order. The minimum distance between successive top-50 T7 transcribed genes was expressed as the number of non-transcribed genes located between transcribed genes. An average minimal distance of 3.35 non-transcribed genes occurred between successive top-50 T7 transcribed genes. The expected average minimal distance determined from 10,000 randomized PBCV-1 genomes was 4.69±0.53. Therefore, our analysis supports the hypothesis that the top-50 T7 transcribed genes are significantly more clustered in the genome than expected by chance (*P* = 0.005).

Several hypotheses might explain this clustering: i) Only a few of the identified immediate-early genes were actively up-regulated at the onset of infection, but the surrounding genes were also identified in the RNA-seq data because of transcriptional read-throughs. ii) Immediate-early expressed genes might be clustered at a few discrete loci as a result of functional constraints, e.g., a specific chromatin structure of the viral genome either at an immediate-early loci or the presence of regulatory sequences controlling the expression of several adjacent genes. While transcriptional read-through occurs in the chloroviruses [15], [18], the second hypothesis of clustering by functional constraints is more speculative. Indeed, we found no statistical support for a global clustering of the orthologs of the top-50 T7 transcribed PBCV-1 genes in the genomes of the ATCV-1 virus that infects *Chlorella heliozoae* SAG [28] and FR483 virus that infects *Micratinium conductrix* Pbi [29] (Table S2). Although these two viruses are related to PBCV-1 and have many genes in common with PBCV-1, their gene orders are highly rearranged relative to PBCV-1 [30]. This finding suggests that the ATCV-1 and FR483 viruses either have different sets of immediate-early genes, which seems unlikely, or that functional constraints common to all chloroviruses is not a valid hypothesis to explain the clustering observed in PBCV-1.

### Cluster Analysis of PBCV-1 Genes

**Figure 3:**
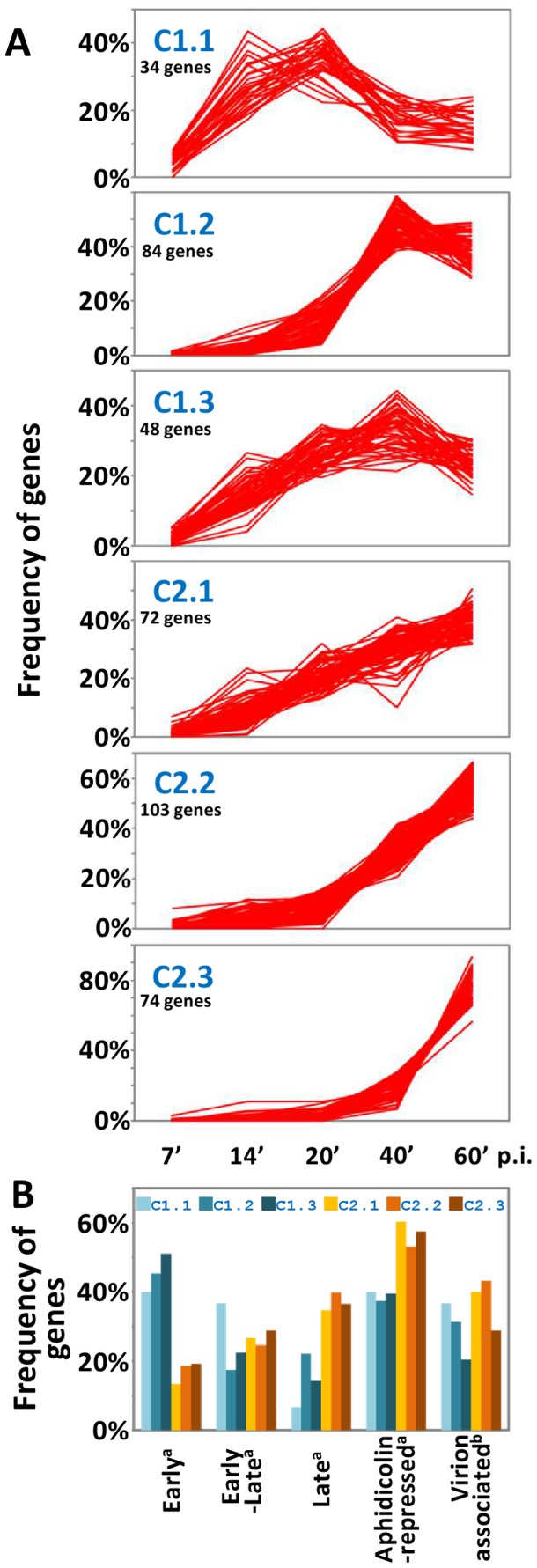
Cluster analysis of PBCV-1 gene expression profiles. (A) Line plot of normalized read counts of PBCV-1 genes for six K-means clusters. (B) Distribution of genes into temporal expression classes and aphidicolin-repressed genes as determined by [18], and virion-associated genes as determined by [6].

K-means cluster analysis was performed to classify genes into six groups based on the similarity of their expression profiles. The six clusters essentially defined two broad temporal mRNA abundance patterns (Fig. 3): i) genes whose transcripts peaked before 60 min p.i. and began to decrease (clusters C1.1, C1.2 and C1.3; collectively referred to as C1.x) and ii) genes with increasing transcript accumulation throughout the first hour of infection (clusters C2.1, C2.2 and C2.3; collectively referred to as C2.x). Differences between C1.1, C1.2 and C1.3 clusters mainly concerned the time of peak expression. Within these clusters, slightly different co-expression patterns were visible. However, the K-means clustering algorithm failed to resolve them into separate groups even when allowing a higher number of K-means clusters. In contrast, variations between C2.x clusters concerned the dynamics of expression: for example genes in cluster C2.1 showed constantly increasing mRNA levels beginning from T7 whereas cluster C2.3 contained genes whose mRNAs appeared at 20 min p.i., and exhibited a sharp increase at 40 min p.i. Gene functional classes did not differ between clusters except genes encoding virion structural proteins (including capsid proteins) were primarily in the C2.x clusters. For example, genes involved in transcription, DNA replication, signaling, sugar metabolism and manipulation were present in every cluster (Table S1).

Clusters obtained from RNA-seq analyses were compared to the three temporal gene classes defined in a previous microarray study [18]. In the microarray study, samples were taken at T0, T20, T40, T60, T120, T240 and T360. Genes expressed before viral DNA synthesis begins (60 min p.i.) were classified as early and genes expressed after DNA synthesis begins were classified as late. Some genes that were expressed before DNA synthesis begins, but their mRNAs were still present after 60 min p.i., were classified as early-late. In the microarray study, the genes classified as early were not detected after 60 min p.i. In the current study, gene products in the C1.x clusters were decreasing by 60 min p.i. As expected, C1.x clusters contained a higher proportion of early genes whereas C2.x clusters contained a higher proportion of late genes as well as genes whose expression was inhibited by aphidicolin, an inhibitor of DNA replication [18]. There was no significant difference in the frequencies of genes belonging to the early-late category between C1.x and C2.x clusters, except that C1.1 contained a higher proportion of early-late genes (12/34 = 35%).

A proteome study of the PBCV-1 virion reported that 62% of late-gene encoded proteins defined in the microarray experiments were detected in the mature virion while early and early-late genes encoded 9% and 29% of the virion-associated proteins, respectively [6]. Many virion-associated gene products were also present in C2.x clusters but this bias was less strong than in the temporal classes defined by the microarray analysis: overall C2.x clusters contained 40% (100/249) of the virion associated genes vs. 28% (47/166) for C1.x clusters. A possible reason for the discrepancy between microarray-based temporal gene classes and RNA-seq clusters is that gene expression profiles acquired over only the early stage does not allow fine grouping of genes according to latter temporal classes (i.e., early-late and late genes). Furthermore, the methods used for normalizing expression values differed between the microarray study and the current study. In the microarray study, the reference sample used for normalization consisted of transcripts obtained by mixing equal amounts of total RNA from all 7 time points (20 to 360 min), while in this study we used the sum of reads mapped to the host and virus genomes at each time point to correct for sequencing depth. It is also worth noting that the current procedure is much more sensitive for detecting mRNAs than microarrays. The current results certainly indicate that initiation of PBCV-1 transcripts occurred at different times during the first hour of infection. However, the regulation was not as tight as the microarray experiments implied. Another issue is that virus infection of the host may not be as synchronous as previously thought.

The rate of total RNA synthesis decreased in *C. variabilis* cells after PBCV-1 infection [12]. Unfortunately no internal normalization method is available to handle the possible bias induced by this global decrease. For a majority of the genes, the expression profiles obtained from microarray and RNA-seq experiments positively correlated over the 3 time points shared by both studies (T20, T40 and T60; see Fig. S2). However 37% of the genes had negatively correlated expression profiles. Thus, our results suggest that accurate profiling of PBCV-1 gene transcription in infected *C. variabilis* cells using an RNA-seq approach requires the development of normalization methods specifically tailored for virus infection studies. In the current study we assumed that a constant mRNA pool size, existed; however, this assumption may not occur. The possible change in mRNA pool sizes is a common problem in studying virus infections.

### PBCV-1 Polyadenylation Signal

Using reads that did not match (over their full length) in the initial mapping step, we performed BLAT searches on the reference genome to identify reads that only have one extremity aligned with the reference sequence, while the other extremity is unmatched and contains a poly(A) tract on the 3′ side or a poly(T) tract on the 5′ side. The rationale behind this selection is that these partially-matched reads are expected to overlap with the poly(A) tails of mRNA. The predicted site of pre-mRNA cleavage and poly(A) tail addition was designated as the last matching nucleotide of the read before the unmatched poly(A) or poly(T) tract. To avoid false positives due to sequencing errors, we only considered potential cleavage sites (CS) validated by at least two independent reads (see Material and Methods and Fig. S3). The same analysis was also performed on the host genome as a control.

Using this approach, we identified 36 and 300 potential CS in PBCV-1 and *C. variabilis*, respectively (Tables S3 and S4). The number of detected potential CS is relatively small considering the effective numbers of transcribed mRNAs in both biological entities and the sequencing depth. Furthermore, poly(A+)-containing mRNAs were specifically selected on oligo-dT beads, which rules out the possibility that the studied transcripts lack a poly(A) tail. A possible explanation is that reads containing poly(A) tails received low quality scores due to homopolymer sequencing issues, and were discarded during quality filtering at the base calling step.

**Figure 4:**
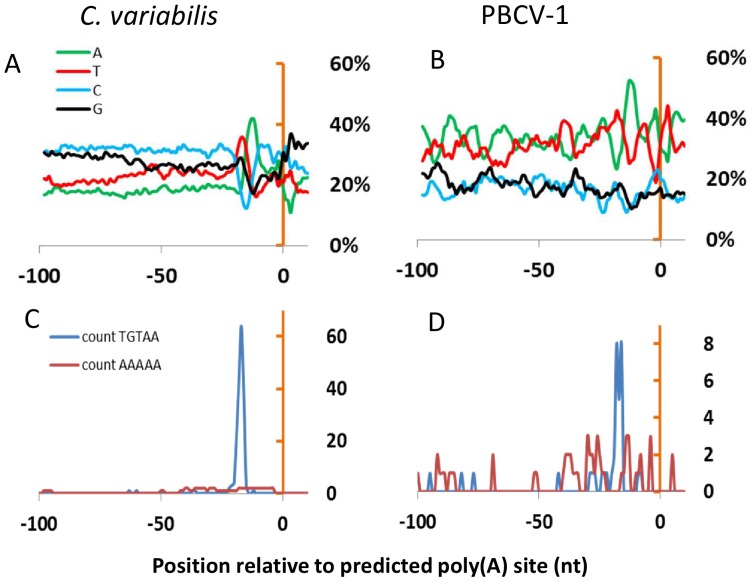
Putative polyadenylation signals in *C. variabilis* and PBCV-1. (A), (B) Nucleotide frequencies within 100 nucleotides upstream from the predicted poly(A+) site in *C. variabilis* and PBCV-1 genes, respectively. Curve profiles were smoothed using the average of 5 neighboring positions. (C), (D) Distribution of putative poly(A+) signals within 100 nucleotides upstream of the predicted poly(A+) site in *C. variabilis* and PBCV-1 genes respectively.

As reported in Fig. 4A and 4B, the nucleotide frequencies around the identified poly(A) sites undergo major changes 10–20 nucleotides upstream from the CSs, indicating a possible concentration of polyadenylation signals in this region. The signal was not as obvious for PBCV-1 because of the smaller number of sequences analyzed, decreasing the signal to noise ratio. Further investigation of the same datasets using sequence logos confirmed the existence of a nucleotide bias in both the virus and host genes between −17 and −10 nucleotides from the CSs, as well as immediately after the CSs (Fig. S4).

To identify possible sequence motifs functioning as a poly(A) signal, pentanucleotide repeats were counted within the 50 nucleotides upstream from the CSs. The 2 most frequent pentanucleotide repeats for *C. variabilis* and PBCV-1, as well as their distribution across the gene 3′ ends are reported in Fig. 4C–D. TGTAA motifs were highly clustered around position −17 in both biological entities. Overall 72% of the examined PBCV-1 gene 3′ extremities (26/36) had a TGTAA motif between positions −24 and −16; in *C. variabilis*, the same motif was present in the same region in 64% of the examined sequences (193/300). In contrast, there was no apparent concentration of AAAAA pentamers around the CSs in either organism although this motif occurred significantly more often than in randomized sequences (Table 2). Such a simple shuffling of DNA sequences is known to overestimate the statistical significance of motifs by neglecting higher-order Markov chain structure, therefore the hypothesis that this motif has a functional role must be taken with caution.

**Table 2:** Number of pentanucleotide words within 50(A) site.

Top words	Observed count	Expect count	Std error	p-value *
**NC64A**				
TGTAA	183	8.7	3.1	0
AAAAA	65	15.9	11.8	1.6 E-05
**PBCV-1**				
TGTAA	29	2.8	1.5	0
AAAAA	28	6.5	4.9	5.7 E-06

* estimated using the Z-score test.

This analysis revealed that, at least a fraction of, PBCV-1 and *C. variabilis* genes probably use the same TGTAA motif as a polyadenylation signal. The TGTAA motif was previously identified as a potential polyadenylation signal in many green algae [31]. Interestingly, all the PBCV-1 genes containing the putative CS were expressed early (Table S3). This bias is not simply due to the fact that we only sequenced RNAs extracted at early time points during infection because virtually all genes were covered by RNA reads at T40 and T60. Further examination of the use of polyadenylation sites in early versus late time points is needed to better characterize the polyadenylation process in PBCV-1. Among NCLDV viruses, mRNA polyadenylation has been most studied in vaccinia virus and mimivirus. The transcription termination of vaccinia virus early genes occurs in response to the sequence TTTTTNT, presumably read by the capping enzyme interacting with the RNA polymerase [32]. This motif did not emerge as a significant sequence signal in our analysis. The 3′-end processing of vaccinia virus intermediate and late transcripts is markedly different and is not associated with specific sequence motifs. In contrast, mimivirus mRNAs from all temporal classes are polyadenylated at a site coinciding exactly with unrelated, but strong palindromic, genomic sequences [33]. Altogether, our results indicate several fundamental differences in the way PBCV-1 and the other NCLDVs handle transcription termination.

### Discovery of Processed Transcripts

Initial annotation of the PBCV-1 genome identified three types of introns: a self-splicing intron in a transcription factor TFIIS-like gene (*a125L*) [34], a spliceosomal-processed intron in a DNA polymerase gene (*a185R*) [35], and a small intron in one of the tRNA genes (Tyr-1) [4]. Reads overlapping with the exon junctions of the two protein genes were readily identified using RNA-seq alignment programs. However, this analysis also identified 133 potential new exon junctions supported by at least 5 distinct read species, revealing putative processed transcripts that were previously unknown (Table S5). Most of these junctions resulted from the excision of a RNA stretch ending with the canonical motifs of donor and acceptor splice sites (i.e., GU and AG) suggesting that they were processed by the spliceosome machinery. These ending dinucleotides were used to infer the direction of transcription of the corresponding DNA region. The potential excision events identified 50 annotated major ORFs dispersed over the entire genome (Red triangles in Fig. 2). Fifteen ORFs contained more than one exon junction (e.g., up to 10 detected exon junctions for *a512R* encoding an unknown protein) suggesting that complex alternative splicing configurations might occur. Ninety-three percent of the excision events resulted in truncated protein products due to either frame shift mutations or shortened ORFs. Furthermore, 27 detected exon junctions occurred on the strand opposite a major gene, indicating that processing occurred in the un-translated region of transcripts originating from a neighboring gene located on the opposite strand or in the anti-sense transcripts of major genes.

Six potential introns were selected for experimental validation using RT-PCR and sequencing (Table S6 and Fig. S5). Predicted cleavage of introns in ORFs A039L, A154L, A181R, and A627R was confirmed by electrophoresis on gels and sequencing. One of the tested introns that existed on the opposite strand to ORF A237R appears to be cleaved as the gel revealed a RT-PCR product with the expected size. However 3 independent attempts to sequence this final product were unsuccessful. The remaining ORF, A604L, did not produce the expected band for the cleaved transcript although the intron prediction was supported by 27 read species containing 209 reads across all time points. However, in all six cases, a RT-PCR product corresponding to the un-cleaved transcripts was obtained. In each of these cases, the un-cleaved transcript was present in higher concentrations than the processed transcript (Fig. S5). This is not surprising because 3 of the transcripts, A039L, A181/182R and A237R encode the functional proteins Skp1, chitinase and homospermidine synthase, respectively [36], [37].

We used the ratio of the number of reads joining two exons (cumulated over all time points) to the MRPN value calculated across the corresponding intron region as a proxy for the frequency of splicing. As expected this ratio was high for A125L and A185R, 97% and 89% respectively, indicating that the efficiency of processing is high for these two known intron-containing genes. In contrast, the ratios for the newly identified processed introns were all ≤33%, with a majority having a ratio <1%. However, experimental quantification of the RNA populations from agarose gels for the five successful validated introns suggests that the RNA-seq-based frequencies are underestimated (Table S6): gel quantification for A039L, A154L, A181R, A237R, and A627R indicated frequencies of 34%, 38%, 55%, 45%, and 40% cleaved transcripts versus 3%, 15%, 11%, 15%, and 15% for RNA-seq based quantification, respectively.

This analysis of RNA-seq data revealed for the first time that a substantial number of PBCV-1 transcripts are processed. However, the biological significance of the excision events is in question because they occur at relatively low frequencies and result in significantly altered protein products or occur in non-coding regions. One possibility is that these splicing events have no functional role and result from cryptic splice signals that are fortuitously recognized by the spliceosome. Alternatively they could reflect a general post-transcriptional regulation process for controlling the number of functional proteins. Significant variations in the ratio of spliced/unspliced transcripts across the time series would support the hypothesis of an active regulation during the early stage, but this is not what we observed. However, we cannot rule out the possibility that significant changes in these ratios occur between the early and late phases of virus replication but we cannot investigate this hypothesis at the present time because the experiment did not span the entire replication cycle.

## Conclusions

This RNA-seq study investigated the dynamic mRNA abundance profiles of both the algal host and virus PBCV-1 during the earliest stages of virus infection. This analysis extends a previous study on PBCV-1 gene transcription that used a microarray approach, which revealed a temporal regulation of PBCV-1 genes [18]. However, the earliest time point in the microarray study was 20 min p.i. and it is clear that many viral mRNAs are being synthesized by that time. In this study we focused on early events and discovered that by 7 min p.i., virus transcripts comprised ∼2% of the poly(A+)-containing RNAs in the cell. At least 50 PBCV-1 genes were transcribed by T7, which seems remarkable for a virus infecting a eukaryotic cell when one considers all the steps required for the virus to initiate infection (see introduction). The RNA-seq approach for studying transcription has several advantages compared to microarrays including a larger dynamic range over which transcripts can be detected. Microarray technologies also suffer from high background levels resulting from cross hybridization and saturation of signals. In contrast, RNA-seq data allow transcripts to be analyzed to a single-base resolution that reveals their precise structure, as for example exon junctions and polyadenylation sites. However, PBCV-1 transcriptome analysis is complicated by overlapping ORFs and the occurrence of mRNAs that read-through adjacent ORFs; these are issues for both procedures. This effect is at least partially mitigated by using MRPN as a measure of transcription levels because its calculation is less affected by possible abnormal values occurring at the extremity of ORFs (i.e., resulting from the accumulation of reads originating from the regular ORF and surrounding read-through mRNAs).

A surprising finding from our study is that in addition to the two PBCV-1 protein-encoding genes previously known to contain an intron, several other PBCV-1 genes produced both spliced and unspliced transcripts. However, no evidence was found that the newly identified splicing events have a functional role. Transcriptome analyses of latter infection stages is required to determine if the relative abundance of spliced transcripts varies over the entire replication cycle, which could suggest active regulation of intron processing. Even if these processed transcripts resulted from ‘spurious’ splicing events promoted by cryptic splicing signals, they can represent up to 55% of the transcripts for a specific gene. This could have an obvious negative consequence on the subsequent number of synthetized proteins.

In summary the current study revealed a rapid activation of viral gene transcription at the onset of infection and the rapid takeover of the host by the virus. By 20 min p.i., transcripts were detected for virtually all PBCV-1 genes and transcript levels continued to increase globally up to 60 min p.i. at which time 41% of the poly(A+)-containing RNAs in the infected cell mapped to the virus genome. For some viral genes, the measured level of transcripts in latter time points (20 to 60 min p.i.) was considerably higher than that of the most highly expressed host nuclear protein genes; e.g., viral gene *a312L* transcripts, encoding a protein of unknown function, are 37 times higher than the most highly expressed host gene at T60. These results also highlight the efficiency of the viral regulatory sequence(s) to promote high transcriptional activity in the *C. variabilis* host.

## Materials and Methods

### Strains, Culture Conditions, and RNA Isolation and Sequencing

*Chlorella variabilis* cells (2×10^7^ cells/ml), actively growing in continuous light (ca. 25 uEi,m^−2^,sec^−1^), were concentrated by centrifugation and re-suspended in fresh MBBM medium to 1.0×10^8^ cells/ml and grown for 1 hr under standard growth conditions (17) to allow the cells to acclimate to fresh medium. For the time 0 (T0) sample, a 30 ml aliquot (3.0×10^9^ cells) of cells was pelleted by 30 sec centrifugation at 10,000 rpm, flash frozen in liquid nitrogen and then virus PBCV-1 was added at an m.o.i. of 5 to the frozen sample; the sample was stored at −80 C. For the remaining time points PBCV-1 was added to the actively growing cells at an m.o.i. of 5; 30 ml of cells were collected at appropriate times by centrifugation, flash frozen in liquid nitrogen and stored at −80 C until use. Growth and purification of PBCV-1 have been described previously [17].

The isolation and processing of RNA from the uninfected and PBCV-1 infected *C. variabilis* cells was also described previously [38]. The RNA sequencing data reported in this paper have been deposited in the NCBI database (Bioproject accession no. PRJNA210187).

### Transcriptome Analysis

Initial alignment, statistics, and clustering analyses have already been described in [38]. For the PBCV-1 targeted analysis described herein, several additional procedures were performed. RNA-seq read mapping was performed in two steps. First, a list of exon junctions for both the host and PBCV-1 using the genome annotations was compiled. This list served to help in the mapping of reads that span over two consecutive exons in the second step of the analysis. *De novo* identification of exon junctions was also performed using the TopHat program [39] to complete the list with possible exon junctions that were missed by gene prediction programs. All RNA-seq datasets were pooled and analyzed together in a single TopHat run. TopHat was set up so that it only considered read alignments with at most 2 mismatches with the target reference sequence and a maximum intron size of 2000 bp. Because TopHat only reports potential exon junctions that result from the removal of introns that end by “GT-AG”, “GC-AG” and “AT-AC” (i.e., spliceosome-processed introns), reads that failed to align with TopHat were further aligned using the Blat program [40] to identify putative exon junctions resulting from spliceosome-independent excisions. In this case, the candidate junctions were identified when two segments from the same read were mapped at two distinct loci separated by less than 2000 bp on the same genomic sequence. For both the TopHat and Blat methods we only considered candidate junctions validated by at least 5 read species to limit false positives. A read species is defined as a group of identical reads irrespective of the strand (i.e., forward and reverse reads belong to the same read species); thus validating read species refer to reads that overlap with the same genomic feature (i.e., in this case an exon junction) with an offset of one or more nucleotides. In a second step, reads from each individual RNA-seq dataset were simultaneously mapped to both the reference genomes and the collection of candidate exon junctions. For subsequent expression analyses, we only considered reads that generated a unique alignment in the reference genomes with at most 2 mismatches. The PBCV-1 genome has inverted repeats at its termini [41] containing ORFs A002bL, A002cR, A002L, A003R and A005R that have strictly identical sequences to ORFs A690fR, A690eR, A691R, A689L and A682L, respectively. To measure the number of reads for these genes we realigned each individual RNA-seq dataset allowing at most two alignments in the reference genomes. The number of reads for each of these genes was divided by two to account for the ambiguity of mapping.

### Read Count Normalization

To correct for gene length, we computed the Median Reads Per Nucleotide (MRPN) that is the median of read coverage values for each nucleotide position in the ORF. The rationale behind this choice is that the median is less affected by extreme and abnormal values than the mean utilized for example in the calculation of Reads Per Kilobase per Million reads (RPKM) [42]. Thus, in addition to being less affected by local sequencing bias, this measure is particularly relevant for PBCV-1 genes where the transcription of genes frequently begins or terminates in neighboring genes, which can artificially and locally inflate read counts per gene. To further correct for sequencing depth, MRPN values for both PBCV-1 and *C. variabilis* ORFs were normalized by a scaling factor equal to the ratio of the sum of reads mapped to the host and virus genomes in the dataset (i.e., T0, T7, T14, T20, T40 and T60) to the sum of reads mapped to the host and virus genomes at T0.

### Sequence and Expression Profile Analyses

K-means clustering was performed with the Cluster 3.0 program [43] using Euclidean distance as similarity metric and setting the number of cluster to 6. Sequence logo was generated by compiling a file containing sequences from position −100 to +10 nucleotides around the predicted poly(A+) sites. This file was analyzed by the WebLogo program [44] using default parameters.

### Intron Processing Validation

RNA was extracted from cells (10^8^ cells/ml) infected with PBCV-1 60 min p.i. (m.o.i of 5) using the Plant Cell RNA isolation kit (Qiagen). DNA traces were removed with the Turbo DNA-Free kit (Ambion, Austin, TX) following the manufacturer’s instructions. Total RNA was quantified with a NanoDrop spectrophotometer (NanoDrop Technologies, Wilmington, DE).

For RT PCR, primers were designed outside of the putative intron fragment and synthesized by In Vitrogen-Life Technologies (Grand Island, NY). Reverse transcription (RT) was carried out using the RevertAid First Strand cDNA Synthesis kit (Fermentas, Vilnius, Lithuania). PCR was conducted with Phusion High-Fidelity DNA Polymerase (New England Biolabs, Beverly, MA). PCR products were resolved by electrophoresis in a 1.8% agarose gel in 1Χ TAE buffer. Resolved DNA fragments were excised from the agarose gel and extracted using the QIAEX II Gel Extraction kit (Qiagen). Sequencing was carried out by Eurofins MWG Operon and Davis Sequencing services (Davis, CA).

The relative quantity of RNA populations was estimated using Quantity One software (Bio-Rad, Hercules, CA). The volume of each band was estimated (intensity area) and the relative quantity was calculated as intensity expressed as percentage of the total intensity of all the bands in a lane.

## Supporting Information

Figure S1 **Relative normalized levels of transcripts in PBCV-1 and** ***Chlorella variabilis.*** (A) PBCV-1 ORFs were grouped into transcript level classes, which are designed by a color code. Transcript accumulation was calculated by means of the MRPN formula and normalized for sequencing depth. (B) Same as (A) for NC64A ORFs.
(PPTX)

Figure S2 **Correlation coefficients between microarray-based and RNA-seq-based expression profiles.** Correlation coefficients were calculated over the time points T20, T40 and T60. Microarray-based expression values were from Yanai-Balser et al. [18].
(PPTX)

Figure S3 **detection of polyadenylation sites.** The figure shows the alignment of 8 RNA-seq reads (Panel A) onto the reference PBCV-1 genome (Panel B). The 8 reads belonged to 3 different read species (3 different segments of the same locus shifted by one or more nucleotides). Stop codon of gene A248R, putative poly(A) signal and poly(A) tail addition site are shown in boxes.
(PPTX)

Figure S4 **sequence logo representation showing nucleotide biases around poly(A) cleavage sites.**
(PPTX)

Figure S5 **Agarose gel electrophoresis of PCR-amplified products representing targeted PBCV-1 transcripts.**
(PPTX)

Table S1 MRPN values for virus and host genes.
(XLSX)

Table S2 Average Minimal Distances between orthologs of top-50 T7 transcribed PBCV-1 genes.
(DOCX)

Table S3 Putative polyadenylation site in PBCV-1 genes.
(DOCX)

Table S4 Putative polyadenylation site in *C. variabilis* genes.
(DOCX)

Table S5 Potential intron cleavage sites in PBCV-1 genes.
(DOCX)

Table S6 PCR amplification of putative excised PBCV-1 transcripts.
(DOCX)
