# Supplementary material for: Deep RNA Sequencing Reveals Hidden Features and Dynamics of Early Gene Transcription in Paramecium bursaria Chlorella Virus 1
Source: PLoS One. 2014 Mar 7;9(3):e90989. doi: 10.1371/journal.pone.0090989 (PMC3946568; doi:10.1371/journal.pone.0090989)
Supplement: Table S2 — Average Minimal Distances between orthologs of top-50 T7 transcribed PBCV-1 genes. (DOCX) [file pone.0090989.s007.docx]

**Table S2**. Average Minimal Distances between orthologs of top-50 T7 transcribed PBCV-1 genes

| Virus | Number of identified orthologs to PBCV-1 T7 transcribed genes | AMD*_obs_ | AMD*_exp_  (average± standard deviation) |
| --- | --- | --- | --- |
| ATCV-1 | 32 | 5.33 | 5.55±0.78 |
| FR483 | 31 | 5.97 | 5.70±0.81 |

*: orthologs were identified using the reciprocal best BLASTP hit criterion

**: Average Minimal Distance
