# Supplementary material for: Deep RNA Sequencing Reveals Hidden Features and Dynamics of Early Gene Transcription in Paramecium bursaria Chlorella Virus 1
Source: PLoS One. 2014 Mar 7;9(3):e90989. doi: 10.1371/journal.pone.0090989 (PMC3946568; doi:10.1371/journal.pone.0090989)
Supplement: Table S3 — Putative polyadenylation site in PBCV-1 genes. (DOCX) [file pone.0090989.s008.docx]

**Table S3**. Putative polyadenylation site in PBCV-1 genes

| Location of cleavage site (CS) | Strand | Location of TGTAA motif relative to CS | Gene | Temporal class | Comment |
| --- | --- | --- | --- | --- | --- |
| 1770 | - | -17 | a004L | unknown |  |
| 1855 | - |  | a004L | unknown |  |
| 23102 | - | -20 | A037L | Early |  |
| 38123 | + | -19 | A067R | Early |  |
| 40441 | - | -16 | A077L | Early |  |
| 50182 | + |  |  |  | strand opposite to A094L |
| 55720 | - | -19 | A103R | Early |  |
| 56690 | - |  | A108bL | unknown |  |
| 60812 | + |  | A111/114R | Early |  |
| 66066 | - | -17 | A125L | Early |  |
| 68995 | - | -16 | A131L | Early |  |
| 78894 | + |  | A153R | Early-Late | within ORF |
| 80207 | - | -20 | A157L | Early-Late |  |
| 81839 | + |  | A161R | Early |  |
| 108123 | - | -16 | A214L | Early |  |
| 125657 | + | -17 | A248R | Early |  |
| 146588 | - | -16 |  |  | strand opposite to A286R |
| 148208 | - | -16 | A289L | Early-Late |  |
| 156004 | - | -18 | A312L | Early-Late |  |
| 156566 | + |  |  |  | strand opposite to A312L |
| 171284 | + | -20 |  |  | strand opposite to A342L |
| 172921 | + | -18 | A348R | Early |  |
| 172990 | + | -60 | A348R | Early |  |
| 198827 | - | -18 | A408L | Early-Late |  |
| 199946 | - | -20 | A410L | Early |  |
| 201819 | + | -19 | A412R | Early |  |
| 211475 | - | -16 | A431L | Early |  |
| 213256 | - | -16 | A440L | Early |  |
| 219421 | - | -16 | A452L | Early |  |
| 233687 | - | -18 | A486L | Early |  |
| 247819 | + | -18 | A512R | Early |  |
| 262996 | - | -21 | A548L | Early |  |
| 299264 | - |  | A623L | Early |  |
| 302413 | - | -20 | A628L | Early |  |
| 318153 | - | -17 | A666L | Early |  |
| 328574 | + |  | A687R | Early |  |
