# Supplementary material for: Deep RNA Sequencing Reveals Hidden Features and Dynamics of Early Gene Transcription in Paramecium bursaria Chlorella Virus 1
Source: PLoS One. 2014 Mar 7;9(3):e90989. doi: 10.1371/journal.pone.0090989 (PMC3946568; doi:10.1371/journal.pone.0090989)
Supplement: Table S4 — Putative polyadenylation site in C. variabilis genes. (DOCX) [file pone.0090989.s009.docx]

**Table S4**. Putative polyadenylation site in *C. variabilis* genes

| Scaffold | Location of cleavage site (CS) | Strand | Location of TGTAA motif relative to CS |
| --- | --- | --- | --- |
| scaffold_1 | 101376 | + |  |
| scaffold_1 | 351939 | + |  |
| scaffold_1 | 462483 | - | -20 |
| scaffold_1 | 506049 | - |  |
| scaffold_1 | 656752 | + | -18 |
| scaffold_1 | 1002637 | + | -17 |
| scaffold_1 | 1099385 | - | -21 |
| scaffold_1 | 1114203 | + | -15 |
| scaffold_1 | 1406783 | + |  |
| scaffold_1 | 1532039 | + | -16 |
| scaffold_1 | 1539528 | + | -17 |
| scaffold_1 | 1704837 | + | -19 |
| scaffold_1 | 1768996 | - | -17 |
| scaffold_1 | 1912930 | - | -18 |
| scaffold_1 | 1965762 | - | -18 |
| scaffold_1 | 2348273 | - | -19 |
| scaffold_1 | 2370911 | + | -19 |
| scaffold_1 | 2426347 | + | -17 |
| scaffold_1 | 2487102 | + | -17 |
| scaffold_1 | 2512753 | - | -22 |
| scaffold_2 | 424170 | - |  |
| scaffold_2 | 804686 | + | -17 |
| scaffold_2 | 949538 | - |  |
| scaffold_2 | 1391599 | + | -17 |
| scaffold_2 | 1417018 | + | -17 |
| scaffold_2 | 1505823 | - |  |
| scaffold_2 | 1526356 | - | -17 |
| scaffold_2 | 1532386 | + | -17 |
| scaffold_2 | 1713041 | + | -19 |
| scaffold_2 | 1979529 | - | -16 |
| scaffold_2 | 2120928 | + | -17 |
| scaffold_2 | 2191953 | + | -18 |
| scaffold_2 | 2279566 | - |  |
| scaffold_2 | 2374287 | + | -16 |
| scaffold_2 | 2418961 | + | -17 |
| scaffold_2 | 2528895 | - |  |
| scaffold_3 | 374667 | + |  |
| scaffold_3 | 447546 | - |  |
| scaffold_3 | 458341 | - |  |
| scaffold_3 | 655600 | + |  |
| scaffold_3 | 677596 | + |  |
| scaffold_3 | 763665 | - | -24 |
| scaffold_3 | 878507 | + | -18 |
| scaffold_3 | 926212 | - | -17 |
| scaffold_3 | 929532 | + | -16 |
| scaffold_3 | 1243290 | + | -16 |
| scaffold_3 | 1280646 |  | -18 |
| scaffold_3 | 1543141 | - | -17 |
| scaffold_3 | 1566037 | + |  |
| scaffold_3 | 1699812 | + |  |
| scaffold_3 | 1738851 | + | -16 |
| scaffold_3 | 1919165 | + | -18 |
| scaffold_3 | 2153467 |  |  |
| scaffold_3 | 2381946 | + | -18 |
| scaffold_4 | 247757 | + |  |
| scaffold_4 | 260917 | - | -20 |
| scaffold_4 | 772970 | + | -19 |
| scaffold_4 | 784933 | - | -17 |
| scaffold_4 | 831056 | - | -17 |
| scaffold_4 | 986620 | - | -16 |
| scaffold_4 | 1291087 | + |  |
| scaffold_4 | 1386729 | + |  |
| scaffold_4 | 1397693 | - | -19 |
| scaffold_4 | 1444214 | + | -18 |
| scaffold_4 | 1563819 | + | -17 |
| scaffold_4 | 1583651 |  |  |
| scaffold_4 | 1585665 | + | -15 |
| scaffold_4 | 1617016 | - | -18 |
| scaffold_4 | 1794697 | - | -18 |
| scaffold_4 | 1887519 | - | -17 |
| scaffold_4 | 1956684 | + | -19 |
| scaffold_4 | 1962886 | - |  |
| scaffold_4 | 2196929 | - | -18 |
| scaffold_4 | 2201081 | - | -19 |
| scaffold_5 | 244021 | - | -19 |
| scaffold_5 | 342232 | - | -17 |
| scaffold_5 | 359993 | - | -17 |
| scaffold_5 | 540245 | - | -15 |
| scaffold_5 | 707964 | + | -17 |
| scaffold_5 | 811546 | - |  |
| scaffold_5 | 839028 | - |  |
| scaffold_5 | 1068450 | - | -17 |
| scaffold_5 | 1199736 | - |  |
| scaffold_5 | 1426245 | - | -16 |
| scaffold_5 | 1472377 | - | -16 |
| scaffold_5 | 1844938 | + | -18 |
| scaffold_5 | 1879129 | - |  |
| scaffold_5 | 2062619 | - | -18 |
| scaffold_5 | 2178051 | - |  |
| scaffold_6 | 99124 | + | -19 |
| scaffold_6 | 399144 | + | -18 |
| scaffold_6 | 782002 | - | -18 |
| scaffold_6 | 1105550 | - | -19 |
| scaffold_6 | 1120746 |  |  |
| scaffold_6 | 1169207 | + | -16 |
| scaffold_6 | 1269032 | - | -16 |
| scaffold_6 | 1435182 | - | -16 |
| scaffold_6 | 1626861 | + | -17 |
| scaffold_6 | 1902565 | + | -16 |
| scaffold_6 | 1928786 | + | -17 |
| scaffold_6 | 2056299 | + | -18 |
| scaffold_7 | 1014517 | + | -17 |
| scaffold_7 | 1211986 | + | -18 |
| scaffold_7 | 1288118 | - | -18 |
| scaffold_7 | 1361843 | + |  |
| scaffold_7 | 1430556 | - | -17 |
| scaffold_7 | 1490041 | + |  |
| scaffold_7 | 1495502 | - | -18 |
| scaffold_7 | 1511102 | + | -17 |
| scaffold_7 | 1621702 | - | -18 |
| scaffold_7 | 1689140 | - | -16 |
| scaffold_8 | 418841 | - | -16 |
| scaffold_8 | 523507 | - |  |
| scaffold_8 | 603471 | + | -17 |
| scaffold_8 | 753153 | - | -18 |
| scaffold_8 | 1058575 | + |  |
| scaffold_8 | 1063710 | + |  |
| scaffold_8 | 1112638 | + | -18 |
| scaffold_8 | 1249494 | + |  |
| scaffold_8 | 1363470 | + |  |
| scaffold_8 | 1454143 | - | -17 |
| scaffold_8 | 1552983 | - | -17 |
| scaffold_8 | 1701621 | - | -17 |
| scaffold_8 | 1751106 | - |  |
| scaffold_8 | 1765344 | - |  |
| scaffold_9 | 261355 | + | -17 |
| scaffold_9 | 514339 | + | -19 |
| scaffold_9 | 570490 | + |  |
| scaffold_9 | 581887 | - | -17 |
| scaffold_9 | 609029 | + | -18 |
| scaffold_9 | 735963 | + |  |
| scaffold_9 | 741436 | + | 43 |
| scaffold_9 | 1152003 | - | -17 |
| scaffold_9 | 1152192 | + |  |
| scaffold_9 | 1414339 | - | -18 |
| scaffold_9 | 1432494 | - | -17 |
| scaffold_9 | 1515664 | + | -17 |
| scaffold_9 | 1516370 | + | -18 |
| scaffold_9 | 1609449 | - |  |
| scaffold_10 | 651941 | + |  |
| scaffold_10 | 652022 | + | 6 |
| scaffold_10 | 1090523 | - | -17 |
| scaffold_10 | 1236728 | - | -17 |
| scaffold_11 | 76827 | + | -19 |
| scaffold_11 | 320335 | + | -18 |
| scaffold_11 | 385210 | + |  |
| scaffold_11 | 386979 | + |  |
| scaffold_11 | 625627 | + |  |
| scaffold_11 | 992048 | + | -16 |
| scaffold_11 | 1005236 | - | -17 |
| scaffold_11 | 1128266 | + |  |
| scaffold_11 | 1276159 | + |  |
| scaffold_12 | 96216 | - |  |
| scaffold_12 | 100323 | + | -17 |
| scaffold_12 | 125646 | - | -20 |
| scaffold_12 | 133249 | - |  |
| scaffold_12 | 589957 | + | -17 |
| scaffold_12 | 620419 | + | -16 |
| scaffold_12 | 673419 | - | -18 |
| scaffold_12 | 960030 | - | -17 |
| scaffold_12 | 1088507 | - | -16 |
| scaffold_12 | 1127099 | + |  |
| scaffold_13 | 254790 | - |  |
| scaffold_13 | 618318 | - | -16 |
| scaffold_13 | 688526 | + |  |
| scaffold_13 | 792915 | - | -19 |
| scaffold_13 | 865502 | - | -16 |
| scaffold_13 | 958657 | - |  |
| scaffold_13 | 1111136 | - | -19 |
| scaffold_13 | 1204279 | + | -17 |
| scaffold_13 | 1209405 | + |  |
| scaffold_14 | 307438 | - | -18 |
| scaffold_14 | 721490 | + |  |
| scaffold_14 | 828799 | - | -17 |
| scaffold_14 | 872316 | + | -17 |
| scaffold_14 | 1092044 | + | -19 |
| scaffold_14 | 1195049 | + | -17 |
| scaffold_14 | 1230857 | + |  |
| scaffold_14 | 1255205 | - | -18 |
| scaffold_15 | 434919 | - | -17 |
| scaffold_15 | 475784 | - | -17 |
| scaffold_15 | 603494 | - | -16 |
| scaffold_15 | 635742 | - | -18 |
| scaffold_15 | 872270 | - |  |
| scaffold_15 | 1010223 | - | -17 |
| scaffold_15 | 1054793 | - |  |
| scaffold_15 | 1111705 | - | -96 |
| scaffold_15 | 1143111 | + | -18 |
| scaffold_15 | 1230143 | - | -17 |
| scaffold_16 | 213733 | + |  |
| scaffold_16 | 251912 | - |  |
| scaffold_16 | 839831 | - |  |
| scaffold_17 | 382353 | - | -16 |
| scaffold_17 | 396640 | + | -16 |
| scaffold_17 | 645019 | - |  |
| scaffold_18 | 189937 | - | -16 |
| scaffold_18 | 225393 | - | -16 |
| scaffold_18 | 593204 | + | -16 |
| scaffold_18 | 638095 | - | -17 |
| scaffold_18 | 699418 | + | -18 |
| scaffold_18 | 833083 | - |  |
| scaffold_18 | 845609 | + | -18 |
| scaffold_18 | 895845 | + | -17 |
| scaffold_18 | 938142 | + | -18 |
| scaffold_18 | 949054 | + | -16 |
| scaffold_18 | 962999 | - |  |
| scaffold_18 | 1064663 | + |  |
| scaffold_18 | 1118358 | - | -18 |
| scaffold_19 | 178958 | - | -19 |
| scaffold_19 | 448163 | - | -17 |
| scaffold_19 | 697370 | + | -16 |
| scaffold_19 | 987732 | + |  |
| scaffold_19 | 1042678 | + | -16 |
| scaffold_20 | 189811 | + |  |
| scaffold_20 | 322763 | - |  |
| scaffold_20 | 413766 | - | -17 |
| scaffold_20 | 435353 | + | -16 |
| scaffold_20 | 651080 | + |  |
| scaffold_20 | 700201 | - | -18 |
| scaffold_20 | 864921 | + |  |
| scaffold_21 | 197575 | + | -16 |
| scaffold_21 | 332711 | - |  |
| scaffold_21 | 479637 | + | -18 |
| scaffold_21 | 569820 | - |  |
| scaffold_21 | 579670 | - |  |
| scaffold_21 | 835095 | + | -16 |
| scaffold_22 | 28396 | - | -18 |
| scaffold_22 | 370884 | + | -19 |
| scaffold_22 | 395958 | + | -17 |
| scaffold_22 | 536974 | - |  |
| scaffold_22 | 600803 | + |  |
| scaffold_22 | 684388 | - |  |
| scaffold_22 | 869531 | - | -17 |
| scaffold_23 | 349409 | + |  |
| scaffold_23 | 420067 | - | -19 |
| scaffold_23 | 771562 | - | -19 |
| scaffold_24 | 288229 | - |  |
| scaffold_24 | 306931 | + | -17 |
| scaffold_24 | 324965 | + |  |
| scaffold_24 | 419088 | - | -18 |
| scaffold_24 | 490627 | + | -19 |
| scaffold_24 | 549312 | + | -17 |
| scaffold_24 | 595309 | + |  |
| scaffold_24 | 616000 | + |  |
| scaffold_24 | 631049 | + |  |
| scaffold_24 | 641871 | + | -18 |
| scaffold_24 | 713355 | - |  |
| scaffold_25 | 263888 |  |  |
| scaffold_25 | 328059 | - |  |
| scaffold_25 | 470676 | - | -16 |
| scaffold_26 | 139253 | - | -18 |
| scaffold_26 | 141313 | + | -16 |
| scaffold_26 | 154336 | + | -17 |
| scaffold_26 | 168073 | - |  |
| scaffold_26 | 184587 |  | -19 |
| scaffold_26 | 184685 | + | -18 |
| scaffold_26 | 217503 | + | -18 |
| scaffold_26 | 229443 | - | -22 |
| scaffold_26 | 231926 | - |  |
| scaffold_26 | 235938 |  |  |
| scaffold_26 | 259208 | - | -18 |
| scaffold_26 | 259636 | + |  |
| scaffold_26 | 405214 | + | -20 |
| scaffold_26 | 434778 | + | -17 |
| scaffold_26 | 437584 | - | -17 |
| scaffold_26 | 490841 | + | -17 |
| scaffold_26 | 506007 | - | -21 |
| scaffold_27 | 182144 | + | -16 |
| scaffold_28 | 227024 | + | -17 |
| scaffold_28 | 321964 | - |  |
| scaffold_29 | 218540 | - | -19 |
| scaffold_29 | 233056 | + | -16 |
| scaffold_29 | 294653 | + | -21 |
| scaffold_29 | 297481 | - |  |
| scaffold_29 | 345724 | + | -17 |
| scaffold_29 | 347382 | + | -17 |
| scaffold_31 | 439352 | - |  |
| scaffold_32 | 130185 | + | -18 |
| scaffold_33 | 157911 | - |  |
| scaffold_33 | 158953 | + | -16 |
| scaffold_34 | 189931 | - | -17 |
| scaffold_35 | 141432 | + | -19 |
| scaffold_35 | 181961 | + |  |
| scaffold_35 | 264881 | + | -20 |
| scaffold_35 | 301711 | + |  |
| scaffold_37 | 251667 | - | -19 |
| scaffold_37 | 256929 | + | 24 |
| scaffold_37 | 257057 | - |  |
| scaffold_37 | 279799 | + | -16 |
| scaffold_38 | 137419 | - | -20 |
| scaffold_38 | 213770 | - | -19 |
| scaffold_42 | 80486 | + | -16 |
| scaffold_44 | 5031 | + | -16 |
| scaffold_67 | 3672 | - |  |
| scaffold_67 | 4309 | - | -60 |
| scaffold_67 | 5394 | - |  |
| scaffold_67 | 7544 | - |  |
| scaffold_67 | 11705 | - | -65 |
| scaffold_67 | 12288 | - |  |
| scaffold_70 | 6430 |  |  |
