# Supplementary material for: Deep RNA Sequencing Reveals Hidden Features and Dynamics of Early Gene Transcription in Paramecium bursaria Chlorella Virus 1
Source: PLoS One. 2014 Mar 7;9(3):e90989. doi: 10.1371/journal.pone.0090989 (PMC3946568; doi:10.1371/journal.pone.0090989)
Supplement: Table S5 — Potential intron cleavage sites in PBCV-1 genes. (DOCX) [file pone.0090989.s010.docx]

Table S5: Potential intron cleavage sites in PBCV-1 genes.

| **Junction location** | | **Donnor\|acceptor splice sites** | **Inferred strand of DNA template** | **Recipient gene** | **Nb read species overlaping exon junction** | **Number of read overlaping exon junction** | | | | |  | **MNRC across intron** | | | | | **Splicing efficiency ratio** |
| --- | --- | --- | --- | --- | --- | --- | --- | --- | --- | --- | --- | --- | --- | --- | --- | --- | --- |
| **Left exon last base** | **Right exon first base** |  |  |  |  | **T7** | **T14** | **T20** | **T40** | **T60** |  | **T7** | **T14** | **T20** | **T40** | **T60** |  |
| 66,228 | 66,629 | TCA\|TTA | **L** | A125L | 31 | 0 | 10 | 178 | 1496 | 997 |  | 0 | 3 | 24 | 31 | 27 | 96.9% |
| 96,593 | 96,695 | GGT\|AGT | **F** | A185R | 29 | 4 | 58 | 341 | 978 | 574 |  | 0 | 2 | 10 | 60 | 182 | 88.5% |
| 92,346 | 92,624 | GGT\|AGG | **F** | A181/182R | 27 | 0 | 19 | 69 | 64 | 53 |  | 8 | 78 | 106 | 102 | 119 | 33.2% |
| 19,037 | 19,171 | CCT\|ACA | **R** | A025/027/029L | 11 | 0 | 1 | 0 | 12 | 8 |  | 0 | 0 | 2 | 18 | 27 | 30.9% |
| 92,131 | 92,286 | GGT\|AGC | **F** | A181/182R | 21 | 1 | 10 | 18 | 20 | 13 |  | 3 | 40 | 74 | 77 | 74 | 18.8% |
| 270,035 | 270,128 | CCT\|ACC | **R** | A561L | 20 | 0 | 0 | 1 | 27 | 47 |  | 0 | 1 | 15 | 101 | 252 | 16.9% |
| 92,158 | 92,286 | GGT\|AGC | **F** | A181/182R | 23 | 0 | 3 | 16 | 19 | 10 |  | 3 | 44 | 71 | 80 | 79 | 14.8% |
| 49,047 | 49,174 | TCT\|ACC | **R** | A092/093L | 5 | 0 | 0 | 3 | 3 | 1 |  | 0 | 5 | 12 | 14 | 12 | 14.1% |
| 79,361 | 79,463 | GCT\|ACC | **R** | A154L | 31 | 1 | 109 | 174 | 157 | 158 |  | 173 | 825 | 876 | 963 | 898 | 13.8% |
| 301,923 | 302,041 | GGT\|AGG | **F** | A627R | 26 | 0 | 1 | 9 | 33 | 30 |  | 0 | 9 | 93 | 207 | 172 | 13.2% |
| 22,657 | 22,777 | CCT\|ACC | **R** | A035L | 20 | 0 | 0 | 1 | 22 | 45 |  | 0 | 3 | 61 | 219 | 203 | 12.3% |
| 289,740 | 289,880 | CCT\|ACC | **R** | A604L | 27 | 1 | 18 | 38 | 81 | 71 |  | 59 | 356 | 499 | 356 | 279 | 11.9% |
| 178,497 | 178,639 | GGT\|AGA | **F** | A363R | 5 | 0 | 0 | 2 | 1 | 2 |  | 0 | 2 | 11 | 10 | 16 | 11.4% |
| 102,131 | 102,386 | TCT\|ACC | **R** | A196L | 5 | 0 | 0 | 2 | 4 | 1 |  | 0 | 2 | 10 | 21 | 23 | 11.1% |
| 325,782 | 325,888 | CCT\|ACC | **R** | A682L | 16 | 0 | 1 | 21 | 73 | 66 |  | 0 | 28 | 264 | 629 | 423 | 10.7% |
| 11,192 | 11,355 | GGT\|AGG | **F** | A014R | 6 | 0 | 0 | 0 | 4 | 2 |  | 0 | 1 | 5 | 15 | 30 | 10.6% |
| 171,606 | 171,787 | CCT\|ACC | **R** | A342L | 21 | 0 | 15 | 31 | 56 | 46 |  | 31 | 188 | 328 | 403 | 326 | 10.4% |
| 45,047 | 45,165 | GGT\|AGG | **F** | A085R | 8 | 0 | 0 | 2 | 4 | 4 |  | 1 | 16 | 40 | 39 | 48 | 6.5% |
| 329,423 | 329,532 | GGT\|AGA | **F** | A690eR | 14 | 0 | 1 | 5 | 8 | 9 |  | 11 | 54 | 90 | 95 | 84 | 6.5% |
| 1,274 | 1,383 | TCT\|ACC | **R** | A002bL | 14 | 0 | 1 | 5 | 8 | 9 |  | 11 | 54 | 90 | 95 | 84 | 6.5% |
| 214,914 | 215,211 | GGT\|AGA | **F** | A443R | 7 | 0 | 0 | 0 | 3 | 5 |  | 0 | 11 | 27 | 39 | 42 | 6.3% |
| 325,798 | 325,888 | TCT\|ACC | **R** | A682L | 22 | 0 | 1 | 2 | 38 | 33 |  | 0 | 27 | 264 | 623 | 421 | 5.3% |
| 325,798 | 325,888 | TCT\|ACC | **R** | A682L | 22 | 0 | 1 | 2 | 38 | 33 |  | 0 | 27 | 264 | 623 | 421 | 5.3% |
| 18,905 | 19,038 | CCT\|ACC | **R** | A025/027/029L | 5 | 0 | 0 | 0 | 2 | 4 |  | 0 | 0 | 3 | 45 | 62 | 5.2% |
| 4,535 | 4,635 | CCT\|ACC | **R** | A007/008L | 9 | 0 | 0 | 4 | 13 | 24 |  | 1 | 8 | 31 | 239 | 506 | 5.0% |
| 143,047 | 143,423 | CCT\|ACT | **R** | A278L | 23 | 0 | 0 | 2 | 36 | 18 |  | 0 | 4 | 73 | 585 | 495 | 4.6% |
| **Junction location** | | **Donnor\|acceptor splice sites** | **Inferred strand of DNA template** | **Recipient gene** | **Nb read species overlaping exon junction** | **Number of read overlaping exon junction** | | | | |  | **MNRC across intron** | | | | | **Splicing efficiency ratio** |
| **Left exon last base** | **Right exon first base** |  |  |  |  | **T7** | **T14** | **T20** | **T40** | **T60** |  | **T7** | **T14** | **T20** | **T40** | **T60** |  |
| 282,985 | 283,309 | TCT\|ACC | **R** | A583L | 9 | 0 | 0 | 0 | 10 | 5 |  | 2 | 67 | 91 | 113 | 101 | 3.9% |
| 170,683 | 170,884 | TCT\|ACC | **R** | A342L | 19 | 0 | 4 | 6 | 11 | 14 |  | 16 | 126 | 219 | 328 | 254 | 3.6% |
| 5,866 | 6,103 | GGT\|AGA | **F** | A010R | 5 | 0 | 0 | 2 | 0 | 4 |  | 0 | 1 | 3 | 41 | 121 | 3.5% |
| 106,178 | 106,418 | GGT\|AGC | **F** | A207R | 8 | 0 | 0 | 1 | 1 | 6 |  | 2 | 42 | 62 | 75 | 82 | 3.0% |
| 214,694 | 214,915 | GGT\|AGG | **F** | A443R | 5 | 0 | 0 | 0 | 3 | 5 |  | 0 | 11 | 58 | 110 | 87 | 2.9% |
| 282,423 | 282,658 | CCT\|ACC | **R** | A583L | 6 | 0 | 0 | 2 | 3 | 4 |  | 2 | 44 | 75 | 84 | 97 | 2.9% |
| 282,985 | 283,469 | TCT\|ACC | **R** | A583L | 9 | 0 | 0 | 1 | 2 | 7 |  | 2 | 59 | 81 | 101 | 94 | 2.9% |
| 23,663 | 23,790 | TCT\|ACC | **R** | A039L | 24 | 0 | 1 | 14 | 64 | 88 |  | 0 | 22 | 430 | 3409 | 2705 | 2.5% |
| 263,318 | 263,720 | CCT\|ACC | **R** | A548L | 28 | 1 | 35 | 80 | 63 | 58 |  | 499 | 3362 | 4206 | 782 | 638 | 2.4% |
| 171,816 | 171,992 | CCT\|ACC | **R** | A342L | 8 | 0 | 1 | 1 | 3 | 6 |  | 18 | 77 | 158 | 146 | 120 | 2.1% |
| 171,894 | 171,992 | ACT\|ACC | **R** | A342L | 7 | 0 | 0 | 1 | 7 | 2 |  | 22 | 76 | 165 | 144 | 115 | 1.9% |
| 292,010 | 292,108 | TCT\|ACA | **R** | A609L | 22 | 0 | 2 | 13 | 48 | 48 |  | 0 | 31 | 472 | 3429 | 2294 | 1.8% |
| 308,785 | 309,178 | GGT\|AGG | **F** | A638R | 14 | 0 | 3 | 4 | 14 | 16 |  | 3 | 18 | 125 | 1188 | 830 | 1.7% |
| 81,769 | 81,938 | GGT\|AGG | **F** | A161R | 18 | 0 | 3 | 8 | 26 | 33 |  | 134 | 747 | 1139 | 1249 | 1010 | 1.6% |
| 228,974 | 229,119 | TCT\|ACC | **R** | A473L | 17 | 0 | 0 | 0 | 11 | 23 |  | 0 | 11 | 178 | 1152 | 785 | 1.6% |
| 6,767 | 6,863 | GGT\|AGG | **F** | A010R | 5 | 0 | 0 | 2 | 1 | 2 |  | 0 | 2 | 4 | 81 | 234 | 1.5% |
| 233,838 | 233,974 | ACT\|ACT | **R** | A484L | 9 | 0 | 2 | 3 | 2 | 4 |  | 28 | 188 | 360 | 144 | 80 | 1.4% |
| 221,331 | 221,464 | TCT\|ACC | **R** | A456L | 14 | 0 | 2 | 6 | 14 | 11 |  | 20 | 254 | 666 | 691 | 792 | 1.3% |
| 263,351 | 263,720 | TCT\|ACC | **R** | A548L | 27 | 1 | 5 | 23 | 37 | 44 |  | 480 | 3260 | 4179 | 777 | 633 | 1.2% |
| 245,911 | 246,101 | GGT\|AGC | **F** | A512R | 16 | 1 | 4 | 20 | 41 | 31 |  | 259 | 2313 | 4762 | 3881 | 1918 | 0.7% |
| 245,911 | 246,104 | GGT\|AGG | **F** | A512R | 21 | 0 | 4 | 12 | 42 | 37 |  | 258 | 2309 | 4752 | 3874 | 1914 | 0.7% |
| 81,712 | 81,938 | AGT\|AGG | **F** | A161R | 14 | 0 | 0 | 4 | 9 | 18 |  | 149 | 794 | 1210 | 1406 | 1198 | 0.6% |
| 111,994 | 112,177 | GGT\|AGA | **F** | A219/222/226R | 14 | 1 | 4 | 5 | 9 | 9 |  | 244 | 1529 | 2407 | 892 | 659 | 0.5% |
| 23,663 | 23,814 | TCT\|ACC | **R** | A039L | 17 | 0 | 0 | 1 | 12 | 18 |  | 0 | 21 | 419 | 3406 | 2700 | 0.5% |
| 53,986 | 54,135 | GGT\|AGA | **F** | A100R | 20 | 0 | 1 | 3 | 19 | 12 |  | 8 | 66 | 528 | 3525 | 3437 | 0.5% |
| 221,497 | 221,674 | CCT\|ACT | **R** | A456L | 10 | 0 | 1 | 2 | 4 | 7 |  | 31 | 270 | 695 | 1032 | 1134 | 0.4% |
| 70,093 | 70,401 | GGT\|AGA | **F** | A133R | 8 | 0 | 0 | 1 | 4 | 5 |  | 4 | 68 | 338 | 1209 | 816 | 0.4% |
| 304,870 | 305,148 | GGT\|AGG | **F** | A629R | 6 | 0 | 0 | 2 | 6 | 2 |  | 2 | 47 | 484 | 1238 | 820 | 0.4% |
| **Junction location** | | **Donnor\|acceptor splice sites** | **Inferred strand of DNA template** | **Recipient gene** | **Nb read species overlaping exon junction** | **Number of read overlaping exon junction** | | | | |  | **MNRC across intron** | | | | | **Splicing efficiency ratio** |
| **Left exon last base** | **Right exon first base** |  |  |  |  | **T7** | **T14** | **T20** | **T40** | **T60** |  | **T7** | **T14** | **T20** | **T40** | **T60** |  |
| 325,464 | 325,589 | TCT\|ACA | **R** | A682L | 12 | 0 | 0 | 2 | 6 | 7 |  | 2 | 60 | 567 | 2185 | 1473 | 0.3% |
| 111,604 | 111,792 | CGT\|AGT | **F** | A219/222/226R | 11 | 0 | 1 | 3 | 9 | 7 |  | 237 | 1561 | 2528 | 855 | 559 | 0.3% |
| 70,305 | 70,537 | GGT\|AGA | **F** | A133R | 8 | 0 | 1 | 1 | 2 | 5 |  | 5 | 65 | 339 | 1294 | 903 | 0.3% |
| 246,259 | 246,641 | GGT\|AGA | **F** | A512R | 19 | 0 | 3 | 5 | 13 | 23 |  | 254 | 2113 | 4408 | 3932 | 2212 | 0.3% |
| 77,481 | 77,663 | GGT\|AGG | **F** | A151R | 7 | 0 | 0 | 0 | 5 | 6 |  | 0 | 15 | 223 | 1329 | 1712 | 0.3% |
| 51,591 | 51,792 | GGT\|AGA | **F** | A098R | 13 | 0 | 1 | 3 | 14 | 8 |  | 201 | 1389 | 2893 | 2352 | 1064 | 0.3% |
| 266,858 | 267,048 | ACT\|ACC | **R** | A554/556/557L | 8 | 0 | 0 | 4 | 2 | 3 |  | 59 | 633 | 1125 | 523 | 430 | 0.3% |
| 50,989 | 51,091 | GGT\|AGA | **F** | A098R | 9 | 0 | 1 | 2 | 5 | 7 |  | 138 | 971 | 1806 | 1244 | 652 | 0.3% |
| 194,974 | 195,179 | GGT\|AGG | **F** | A401R | 9 | 0 | 0 | 0 | 8 | 7 |  | 0 | 0 | 14 | 690 | 4283 | 0.3% |
| 221,779 | 221,992 | ACT\|ACC | **R** | A456L | 7 | 0 | 0 | 1 | 5 | 3 |  | 32 | 310 | 753 | 967 | 986 | 0.3% |
| 245,362 | 245,674 | GGT\|AGC | **F** | A512R | 16 | 0 | 0 | 1 | 10 | 17 |  | 238 | 2011 | 4279 | 3124 | 1541 | 0.2% |
| 308,209 | 308,528 | GGT\|AGA | **F** | A638R | 5 | 0 | 0 | 2 | 1 | 3 |  | 4 | 19 | 178 | 1375 | 833 | 0.2% |
| 51,591 | 51,695 | GGT\|AGG | **F** | A098R | 10 | 0 | 4 | 0 | 10 | 5 |  | 186 | 1351 | 2815 | 2500 | 1095 | 0.2% |
| 221,900 | 221,992 | CCT\|ACC | **R** | A456L | 6 | 0 | 1 | 0 | 1 | 4 |  | 25 | 275 | 647 | 773 | 852 | 0.2% |
| 221,331 | 221,441 | TCT\|ACC | **R** | A456L | 7 | 0 | 1 | 0 | 5 | 1 |  | 24 | 296 | 777 | 911 | 1011 | 0.2% |
| 111,994 | 112,161 | GGT\|AGA | **F** | A219/222/226R | 7 | 0 | 0 | 1 | 5 | 7 |  | 246 | 1552 | 2438 | 900 | 672 | 0.2% |
| 95,438 | 95,594 | GGT\|AGG | **F** | A185R | 5 | 0 | 0 | 0 | 1 | 6 |  | 2 | 95 | 549 | 1554 | 1088 | 0.2% |
| 221,739 | 221,992 | GCT\|ACC | **R** | A456L | 5 | 0 | 0 | 1 | 3 | 3 |  | 34 | 324 | 792 | 1039 | 1104 | 0.2% |
| 70,093 | 70,537 | GGT\|AGA | **F** | A133R | 5 | 0 | 0 | 0 | 3 | 2 |  | 4 | 64 | 327 | 1168 | 813 | 0.2% |
| 245,725 | 246,101 | GGT\|AGC | **F** | A512R | 9 | 0 | 0 | 5 | 12 | 13 |  | 274 | 2505 | 5257 | 4305 | 2066 | 0.2% |
| 286,240 | 286,523 | AGT\|AGC | **F** | A593R | 17 | 0 | 1 | 4 | 16 | 20 |  | 782 | 3295 | 4999 | 5783 | 6170 | 0.2% |
| 50,989 | 51,101 | GGT\|AGG | **F** | A098R | 8 | 0 | 0 | 1 | 5 | 3 |  | 137 | 951 | 1764 | 1227 | 649 | 0.2% |
| 289,259 | 289,378 | GGT\|AGC | **F** | a603R | 14 | 0 | 0 | 1 | 5 | 16 |  | 137 | 567 | 949 | 3733 | 6609 | 0.2% |
| 245,725 | 246,104 | GGT\|AGG | **F** | A512R | 10 | 0 | 1 | 0 | 14 | 9 |  | 274 | 2503 | 5235 | 4302 | 2065 | 0.2% |
| 221,497 | 221,659 | CCT\|ACC | **R** | A456L | 5 | 0 | 0 | 0 | 2 | 3 |  | 31 | 276 | 696 | 1055 | 1155 | 0.2% |
| 2,533 | 2,790 | TGA\|GGG | **F** | A005R | 8 | 1 | 5 | 7 | 2 | 3 |  | 972 | 4094 | 4641 | 1318 | 1021 | 0.1% |
| 52,106 | 52,232 | GGT\|AGG | **F** | A098R | 9 | 0 | 0 | 4 | 3 | 4 |  | 202 | 1387 | 2886 | 2449 | 1173 | 0.1% |
| 245,211 | 245,315 | GGT\|AGT | **F** | A512R | 9 | 0 | 0 | 0 | 8 | 4 |  | 201 | 1874 | 3938 | 3114 | 1693 | 0.1% |
| **Junction location** | | **Donnor\|acceptor splice sites** | **Inferred strand of DNA template** | **Recipient gene** | **Nb read species overlaping exon junction** | **Number of read overlaping exon junction** | | | | |  | **MNRC across intron** | | | | | **Splicing efficiency ratio** |
| **Left exon last base** | **Right exon first base** |  |  |  |  | **T7** | **T14** | **T20** | **T40** | **T60** |  | **T7** | **T14** | **T20** | **T40** | **T60** |  |
| 95,931 | 96,345 | GGT\|AGG | **F** | A185R | 5 | 0 | 0 | 0 | 2 | 3 |  | 4 | 104 | 676 | 2384 | 1459 | 0.1% |
| 106,867 | 106,913 | TGA\|CGG | **F** | A208R | 8 | 0 | 14 | 18 | 19 | 14 |  | 2100 | 10761 | 18157 | 19741 | 10976 | 0.1% |
| 137,807 | 138,025 | CCT\|ACC | **R** | A271L | 7 | 0 | 0 | 1 | 1 | 6 |  | 8 | 160 | 670 | 3904 | 3164 | 0.1% |
| 49,891 | 50,005 | TCT\|ACC | **R** | A094L | 12 | 0 | 3 | 7 | 6 | 3 |  | 345 | 2535 | 5255 | 7404 | 4013 | 0.1% |
| 51,591 | 51,852 | GGT\|AGG | **F** | A098R | 7 | 0 | 1 | 1 | 3 | 3 |  | 216 | 1439 | 2968 | 2509 | 1101 | 0.1% |
| 246,847 | 246,937 | GGT\|AGC | **F** | A512R | 11 | 0 | 0 | 2 | 5 | 12 |  | 379 | 3080 | 6211 | 7047 | 3764 | 0.1% |
| 243,458 | 243,582 | CCT\|ACC | **R** | A505L | 5 | 0 | 0 | 3 | 1 | 1 |  | 118 | 1078 | 2283 | 1112 | 859 | 0.1% |
| 245,051 | 245,212 | AGT\|AGG | **F** | A512R | 6 | 0 | 1 | 2 | 3 | 1 |  | 142 | 1322 | 2916 | 2185 | 1162 | 0.1% |
| 52,112 | 52,232 | TGT\|AGG | **F** | A098R | 7 | 0 | 0 | 2 | 4 | 1 |  | 191 | 1387 | 2923 | 2298 | 1087 | 0.1% |
| 51,354 | 51,512 | TGT\|AGC | **F** | A098R | 8 | 0 | 0 | 3 | 3 | 2 |  | 258 | 2004 | 3851 | 3551 | 1508 | 0.1% |
| 51,591 | 51,777 | GGT\|AGC | **F** | A098R | 5 | 0 | 0 | 0 | 4 | 1 |  | 201 | 1382 | 2876 | 2304 | 1058 | 0.1% |
| 263,832 | 263,950 | CCT\|ACC | **R** | A548L | 5 | 0 | 1 | 2 | 3 | 0 |  | 492 | 3362 | 4198 | 855 | 613 | 0.1% |
| 245,362 | 245,680 | GGT\|AGC | **F** | A512R | 6 | 0 | 0 | 1 | 5 | 0 |  | 238 | 2010 | 4272 | 3186 | 1550 | 0.1% |
| 55,071 | 55,324 | GGT\|AGC | **F** | A103R | 5 | 0 | 0 | 0 | 2 | 5 |  | 209 | 1055 | 2208 | 5287 | 4604 | 0.1% |
| 31,899 | 32,247 | GGT\|AGT | **F** | A057aR | 6 | 0 | 0 | 0 | 2 | 4 |  | 0 | 25 | 559 | 5807 | 8644 | 0.0% |
| 51,375 | 51,592 | GGT\|AGG | **F** | A098R | 5 | 0 | 0 | 0 | 3 | 2 |  | 278 | 2225 | 4418 | 4197 | 1673 | 0.0% |
| 106,852 | 106,910 | GGC\|AGG | **F** | A208R | 7 | 1 | 4 | 8 | 6 | 2 |  | 2058 | 10212 | 17375 | 18110 | 9996 | 0.0% |
| 106,852 | 106,910 | GGC\|AGG | **F** | A208R | 7 | 1 | 4 | 8 | 6 | 2 |  | 2058 | 10212 | 17375 | 18110 | 9996 | 0.0% |
| 106,897 | 106,964 | TGC\|AGG | **F** | A208R | 7 | 0 | 3 | 7 | 3 | 3 |  | 2162 | 10732 | 18422 | 19784 | 11102 | 0.0% |
| 106,909 | 106,997 | GGC\|AGG | **F** | A208R | 9 | 0 | 2 | 7 | 2 | 0 |  | 2106 | 10526 | 17879 | 18959 | 10668 | 0.0% |
| 106,951 | 107,006 | GGA\|AGG | **F** | A208R | 7 | 0 | 2 | 4 | 3 | 1 |  | 2032 | 10559 | 17335 | 17654 | 10320 | 0.0% |
| 106,930 | 107,006 | GGC\|AGG | **F** | A208R | 6 | 0 | 1 | 2 | 6 | 1 |  | 2122 | 10526 | 17879 | 18436 | 10499 | 0.0% |
| 106,909 | 106,931 | GGC\|AGG | **F** | A208R | 5 | 0 | 1 | 0 | 3 | 3 |  | 2072 | 10376 | 17713 | 20045 | 11243 | 0.0% |
| 86,393 | 86,616 | CCT\|ACC | **R** | opposite to opposite to A575L | 10 | 0 | 0 | 1 | 9 | 3 |  | 0 | 1 | 7 | 46 | 32 | NA |
| 234,047 | 234,223 | ACT\|ACC | **R** | opposite to opposite to A558L | 11 | 0 | 1 | 6 | 7 | 10 |  | 0 | 2 | 4 | 9 | 15 | NA |
| 116,564 | 116,800 | ACT\|ACC | **R** | opposite to opposite to A312L | 11 | 0 | 0 | 2 | 13 | 56 |  | 87 | 624 | 2668 | 19716 | 78625 | NA |
| 75,354 | 75,458 | CCT\|ACA | **R** | opposite to opposite to A168R | 14 | 0 | 0 | 1 | 12 | 12 |  | 1 | 1 | 10 | 40 | 43 | NA |
| 75,579 | 75,683 | CCT\|ACA | **R** | opposite to opposite to A168R | 7 | 0 | 0 | 1 | 2 | 6 |  | 0 | 1 | 6 | 23 | 27 | NA |
| **Junction location** | | **Donnor\|acceptor splice sites** | **Inferred strand of DNA template** | **Recipient gene** | **Nb read species overlaping exon junction** | **Number of read overlaping exon junction** | | | | |  | **MNRC across intron** | | | | | **Splicing efficiency ratio** |
| **Left exon last base** | **Right exon first base** |  |  |  |  | **T7** | **T14** | **T20** | **T40** | **T60** |  | **T7** | **T14** | **T20** | **T40** | **T60** |  |
| 75,179 | 75,458 | CCT\|ACA | **R** | opposite to opposite to A140/145R | 11 | 0 | 0 | 0 | 4 | 10 |  | 0 | 2 | 17 | 20 | 26 | NA |
| 75,354 | 75,683 | CCT\|ACA | **R** | opposite to opposite to A140/145R | 13 | 0 | 0 | 3 | 13 | 9 |  | 0 | 2 | 39 | 51 | 57 | NA |
| 174,994 | 175,250 | GGT\|AGA | **F** | opposite to A488R | 11 | 0 | 0 | 2 | 5 | 7 |  | 4 | 28 | 68 | 63 | 54 | NA |
| 116,658 | 116,800 | TCT\|ACC | **R** | opposite to A485R | 6 | 0 | 1 | 2 | 2 | 3 |  | 109 | 824 | 1582 | 1061 | 595 | NA |
| 150,489 | 150,735 | GGT\|AGG | **F** | opposite to A482R | 5 | 0 | 0 | 0 | 6 | 3 |  | 15 | 30 | 44 | 49 | 59 | NA |
| 235,833 | 235,963 | CCT\|ACC | **R** | opposite to A363R | 7 | 0 | 1 | 1 | 2 | 4 |  | 0 | 3 | 6 | 27 | 57 | NA |
| 171,036 | 171,193 | GGT\|AGG | **F** | opposite to A352L | 7 | 0 | 0 | 1 | 3 | 7 |  | 3 | 49 | 79 | 78 | 52 | NA |
| 74,385 | 74,527 | TCT\|ACC | **R** | opposite to A352L | 10 | 0 | 0 | 1 | 6 | 7 |  | 22 | 340 | 588 | 867 | 1158 | NA |
| 268,106 | 268,447 | GGT\|AGG | **F** | opposite to A350R | 6 | 0 | 1 | 1 | 1 | 4 |  | 4 | 37 | 28 | 31 | 37 | NA |
| 175,341 | 175,711 | CGT\|AGA | **F** | opposite to A342L | 22 | 0 | 4 | 9 | 29 | 39 |  | 20 | 155 | 292 | 394 | 302 | NA |
| 171,036 | 171,205 | GGT\|AGG | **F** | opposite to A342L | 15 | 0 | 1 | 0 | 7 | 18 |  | 23 | 161 | 286 | 374 | 313 | NA |
| 278,456 | 278,718 | GGT\|AGT | **F** | opposite to A342L | 5 | 0 | 1 | 0 | 3 | 4 |  | 23 | 164 | 296 | 403 | 319 | NA |
| 232,937 | 233,049 | TCT\|ACA | **R** | opposite to A342L | 5 | 0 | 0 | 0 | 5 | 1 |  | 21 | 163 | 306 | 409 | 323 | NA |
| 173,426 | 173,678 | CCT\|ACC | **R** | opposite to A295L | 8 | 0 | 0 | 2 | 6 | 3 |  | 0 | 5 | 12 | 37 | 75 | NA |
| 180,729 | 180,955 | CCT\|ACC | **R** | opposite to A237R | 23 | 0 | 1 | 14 | 56 | 35 |  | 21 | 212 | 350 | 315 | 200 | NA |
| 116,839 | 117,008 | GCT\|ACC | **R** | opposite to A237R | 17 | 0 | 0 | 14 | 34 | 25 |  | 21 | 214 | 355 | 317 | 202 | NA |
| 170,763 | 171,037 | GGT\|AGG | **F** | opposite to A237R | 9 | 0 | 0 | 2 | 8 | 4 |  | 8 | 142 | 246 | 278 | 200 | NA |
| 170,763 | 170,942 | GGT\|AGT | **F** | opposite to A237R | 6 | 0 | 0 | 0 | 3 | 3 |  | 21 | 211 | 341 | 292 | 195 | NA |
| 86,393 | 86,499 | CCT\|ACC | **R** | opposite to A140/145R | 9 | 0 | 0 | 0 | 3 | 8 |  | 0 | 1 | 22 | 24 | 28 | NA |
| 74,678 | 75,118 | CCT\|ACC | **R** | opposite to A140/145R | 15 | 0 | 0 | 1 | 12 | 12 |  | 0 | 2 | 40 | 67 | 66 | NA |
| 74,678 | 74,972 | CCT\|ACC | **R** | opposite to A140/145R | 11 | 0 | 0 | 0 | 9 | 7 |  | 0 | 2 | 31 | 64 | 67 | NA |
| 116,553 | 116,800 | CCT\|ACC | **R** | opposite to A140/145R | 7 | 0 | 0 | 0 | 4 | 10 |  | 0 | 1 | 34 | 63 | 60 | NA |
| 156,829 | 156,866 | CGT\|TTG | **F** | opposite to A140/145R | 5 | 0 | 0 | 2 | 0 | 3 |  | 0 | 1 | 24 | 23 | 26 | NA |
