# Supplementary material for: Deep RNA Sequencing Reveals Hidden Features and Dynamics of Early Gene Transcription in Paramecium bursaria Chlorella Virus 1
Source: PLoS One. 2014 Mar 7;9(3):e90989. doi: 10.1371/journal.pone.0090989 (PMC3946568; doi:10.1371/journal.pone.0090989)
Supplement: Table S6 — PCR amplification of putative excised PBCV-1 transcripts. (DOCX) [file pone.0090989.s011.docx]

Table S6: PCR amplification of putative excised PBCV-1 transcripts

| Primer set | Primer sequence | Intron (nt) | Product from a template (nt) | | Relative distribution of RNA population† | Sequencing confirmed |
| --- | --- | --- | --- | --- | --- | --- |
|  |  |  | Full length | Intron excised |  |  |
| A039L I | atatggacttcatgtttgatcta | 126 | 221 | 95 | 66/34 | yes |
|  | ttaataatcttcaattggcac |  |  |  |  |  |
| A039L II | cgttggagacgacgaaccta |  | 345 | 219 |  |  |
|  | ggcaccaaatgagtccacg |  |  |  |  |  |
| A154L I | tgtgtctcgaagtcgacgaagat | 101 | 195 | 94 | 62/38 | yes |
|  | cagcatcgaggtaacgtccatc |  |  |  |  |  |
| A154L II | tggatatggcaacattccgt |  | 439 | 338 |  |  |
|  | atcgaggtaacgtccatctc |  |  |  |  |  |
| A181R I | gaactatccctctatcgagtca | 277 | 370 | 93 | 45/55 | yes |
|  | atagcattgttcactctggcatt |  |  |  |  |  |
| A181R II | cactgctgacagcaataaca |  | 434 | 157 |  |  |
|  | ccattgacataccgttgaatg |  |  |  |  |  |
| A237R I | tcgtggacctcgcgtggtata | 235 | 315 | 80 | 55/45 | no |
|  | tgacgttagagtccgagctgt |  |  |  |  |  |
| A237R II | tggacctcgcgtggtata |  | 358 | 123 |  |  |
|  | gcggcttcgttatacttcc |  |  |  |  |  |
| A604L I | attacaagagaatatactcaa | 139 | 252 | 113 | no intron excision detected | NA |
|  | atccatactactcatgattaa |  |  |  |  |  |
| A604L II | aactgtcgcgtaacaattcc |  | 331 | 192 |  |  |
|  | tctcatgatgagtcgtgaga |  |  |  |  |  |
| A627R I | cgttcgtcatcacaatcacg | 117 | 237 | 120 | 60/40 | yes |
|  | gactgtcttgactgcatatg |  |  |  |  |  |
| II | cgttcgtcatcacaatcacg |  | 263 | 146 |  |  |
|  | accttcgagcatgatgtcac |  |  |  |  |  |

† full length/intron excised
